# Perioperative Standard Oral Nutrition Supplements Versus Immunonutrition in Patients Undergoing Colorectal Resection in an Enhanced Recovery (ERAS) Protocol: A Multicenter Randomized Clinical Trial (SONVI Study)

**DOI:** 10.1097/MD.0000000000003704

**Published:** 2016-05-27

**Authors:** Pedro Moya, Leticia Soriano-Irigaray, Jose Manuel Ramirez, Alessandro Garcea, Olga Blasco, Francisco Javier Blanco, Carlo Brugiotti, Elena Miranda, Antonio Arroyo

**Affiliations:** From the Department of General Surgery (PM, AA), Division of Colorectal Surgery, University General Hospital of Elche, Elche; Department of Hospital Pharmacy (LS-I), University General Hospital of Elche, Elche; Department of General Surgery (JMR), Division of Colorectal Surgery, University Clinic Hospital Lozano Blesa, Zaragoza; Department of General Surgery (AG), Division of Colorectal Surgery, Hospital of Torrevieja, Torrevieja; Department of General Surgery (OB), Division of Colorectal Surgery, Virgen del Puerto Hospital, Plasencia; Department of General Surgery (FJB), Division of Colorectal Surgery, University Hospital of La Ribera, Alzira; Department of General Surgery (CB), Division of Colorectal Surgery, Hospital of Manacor, Manacor; and Department of Anesthesia (EM), University General Hospital of Elche, Elche, Spain.

## Abstract

To compare immunonutrition versus standard high calorie nutrition in patients undergoing elective colorectal resection within an Enhanced Recovery After Surgery (ERAS) program.

Despite progress in recent years in the surgical management of patients with colorectal cancer (ERAS programs), postoperative complications are frequent. Nutritional supplements enriched with immunonutrients have recently been introduced into clinical practice. However, the extent to which the combination of ERAS protocols and immunonutrition benefits patients undergoing colorectal cancer surgery is unknown.

The SONVI study is a prospective, multicenter, randomized trial with 2 parallel treatment groups receiving either the study product (an immune-enhancing feed) or the control supplement (a hypercaloric hypernitrogenous supplement) for 7 days before colorectal resection and 5 days postoperatively.

A total of 264 patients were randomized. At baseline, both groups were comparable in regards to age, sex, surgical risk, comorbidity, and analytical and nutritional parameters. The median length of the postoperative hospital stay was 5 days with no differences between the groups. A decrease in the total number of complications was observed in the immunonutrition group compared with the control group, primarily due to a significant decrease in infectious complications (23.8% vs. 10.7%, *P* = 0.0007). Of the infectious complications, wound infection differed significantly between the groups (16.4% vs. 5.7%, *P* = 0.0008). Other infectious complications were lower in the immunonutrition group but were not statistically significantly different.

The implementation of ERAS protocols including immunonutrient-enriched supplements reduces the complications of patients undergoing colorectal resection.

This study is registered with ClinicalTrial.gov: NCT02393976.

## INTRODUCTION

The introduction of the Enhanced Recovery After Surgery (ERAS) programs in the last decade has led to substantial improvements in the care of patients undergoing surgery on a scheduled basis.^1,2^ Strong evidence, including several clinical trials and a recent Cochrane Library systematic review, demonstrates that adherence to ERAS protocols can minimize morbidity while being cost-effective in shortening the length of stay (LOS) in the hospital following colorectal surgery.^3–8^ However, postoperative complications remain common, which affect the LOS, costs, and income associated with increased mortality.

Infections remain among the major complications that follow colorectal surgery. It is difficult to anticipate when such complications occur because their causes are varied. Furthermore, immunosuppression caused by surgical stress is one of the most important factors in complication development.^9^

In recent years, standard nutritional formulas have been modified by the addition of arginine, omega-3 fatty acids, glutamine, and other components, which may increase immune responses by modulating inflammatory responses or enhancing protein synthesis after surgery. The potential effects of these immunonutrients include reducing infectious and other postoperative complications.

The aim of this study is to examine whether the joint implementation of immunonutrition with an ERAS program improves morbidity, mortality, and LOS compared with classic nutritional supplements. At present, few studies have investigated the role of immunonutrition, specifically in colorectal surgery. To the best of our knowledge, no studies have investigated immunonutrition within an ERAS program.

## METHODS

Patients treated at 6 Spanish hospitals (University Hospital of Elche, University Hospital Lozano Blesa, Hospital of Torrevieja, Marina Alta Hospital, Hospital of Manacor, and Virgen del Puerto Hospital) with a preoperative diagnosis of colorectal cancer between January 2014 and March 2015 were included. All participating hospitals have vast experience in ERAS^10–12^ and are part of the ERAS Spain.

### Study Design

The SONVI study is a prospective multicenter randomized single-blind trial with 2 parallel treatment groups receiving either the study product (an immune-enhancing feed (IEF)-ATEMPERO produced by Vegenat or hypercaloric, high-protein supplement (HHS)-SUPRESSI of Vegenat) for 7 days prior to colorectal resection and for 5 days postoperatively. The patients were randomized using http://www.randomization.com.

### Feeding Regimens

Patients who completed a staging workup and were deemed suitable candidates for colorectal resection were randomized to either the HHS group or IEF group. The contents of each feed are listed in Table 1.

**TABLE 1:**
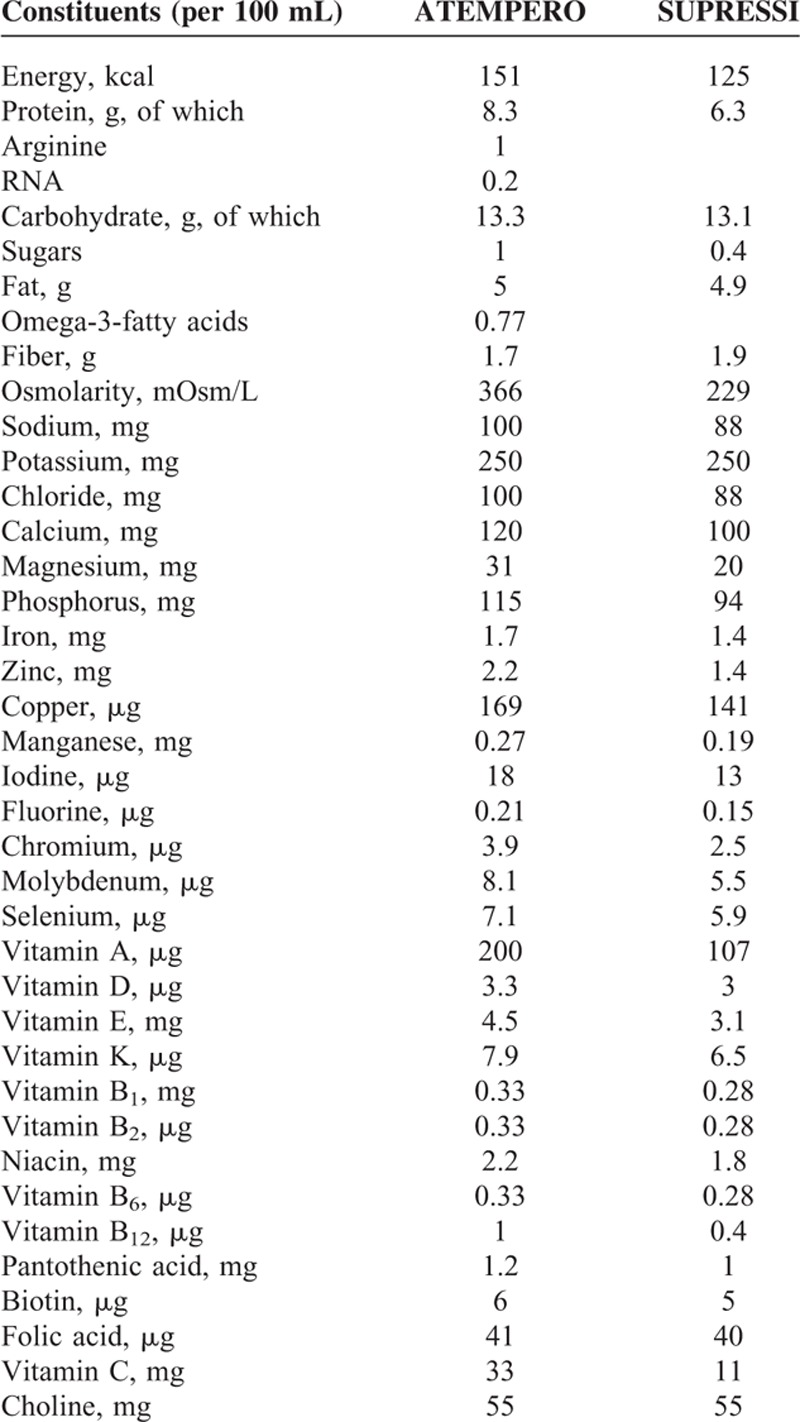
Composition of Diet

The patients were asked to consume 2 cartons (400 mL) of their assigned feed per day for 7 days prior to surgery and to daily record the volume consumed in a dedicated “compliance diary.” This dietary supplement was consumed in addition to normal food intake. No patient received total parenteral nutrition during the preoperative period of the trial. Postoperatively, the patients were asked to consume 2 cartons (400 mL) of either feed each day for 5 days.

## INCLUSION AND EXCLUSION CRITERIA

### Inclusion Criteria

All patients were required to be at least 18 years of age, to be scheduled for surgery for colorectal cancer, to be normo-nourished and to provide written consent.

### Exclusion Criteria

All patients who did not meet the inclusion criteria were excluded. Other exclusion criteria included the need for emergency surgery, an American Society of Anesthesiologists (ASA) physical status IV, renal failure defined via hemodialysis, patients on immunomodulatory or nutritional supplements, hypersensitivity to arginine, omega-3 fatty acids, or nucleotides, the inability to consume oral nutrition (dysphagia, esophageal stricture, and pyloric stenosis), psychiatric disorders, HIV, pregnancy, bowel obstruction, and uncontrolled infection.

### ERAS Protocol

The ERAS interventions used were based on previously published protocols (10–12), which required that, during the preoperative period, the patients be given advice and that they receive intravenous iron supplementation in cases of preoperative anemia and no preparation of the colon (diet low in fiber and enemas before surgery). In all cases, all patients were admitted to surgery day before or the same day. It also required that the patients receive 4 carbohydrate-rich drinks (800 mL) 1 day prior to surgery and 2 additional drinks (400 mL each) on the morning of surgery. During surgery, goal-directed fluids were administered using esophageal Doppler monitoring, hypothermia and drainages were avoided, and epidural anaesthesia used. After surgery, nasogastric tubes were not used; rather, early mobilization was practiced, opioid-free pain control and prophylactic medication for nausea and vomiting used and oral fluids were administered early.

The patients were discharged following the criteria in ERAS. All patients were followed for at least 3 months. An online database was prepared for the collection of data from the various centers.

### Protocol for the Prevention of Surgical Site Infection

All patients were treated according to this protocol and included an antiseptic shower with chlohexidine soap the same day of the intervention, preoperative preparation of the skin with chlohexidine/alcohol solution, intravenous surgical antimicrobial prophylaxis (metronidazole and tobramycin) administered 30 minutes before incision, perioperative glucose levels <200 mg/dL, glove change every 90 minutes, perioperative maintenance of patient normothermia, and no bowel preparation. For extraction of the surgical specimen it was used a self-retractor for laparotomy (Alexis Wound Retractor; Applied Medical, Rancho Santa Margarita, CA or 3 M Steri-Drape Wound Edge Protector, 3 M, Minnesota). The incisions were closed using buried triclosan-coated polydioxanone antimicrobial sutures (PDS Plus Antibacterial Suture; Ethicon Inc, Somerville, NJ); and irrigated with chorexidine solution. The incision were coated with cyanoacrylate tissue adhesive (Dermabond; Ethicon Inc, Somerville, NJ).

### Outcome Measures

Patient baseline characteristics at the time of surgery (age, sex, ASA status, and major comorbidities) were obtained from each patient. ERAS compliance was determined and recorded in the database. Compliance with all interventions was combined and expressed as the percentage of patients who received the correct intervention and documentation.

The 30-day postoperative complications were recorded. Complications were defined as any deviation from the normal postoperative course and divided into minor and major complications. Minor complications included minor risk events, such as wounds infection opened at the bedside, urinary tract infection, or postoperative ileus (Clavien-Dindo I–II).^13^ Major complications included potentially life-threatening complication and those with a need of surgical, endoscopic, or radiological intervention, such as anastomotic leak, abdominal abscess, and pneumonia (Clavien-Dindo III–IV).^13^ Surgical site infection was defined according to the Centers for Disease Control and Prevention classification of surgical site infection and divided into superficial and deep incisional (spontaneous drainage of purulent material from the wound or from the surgeon's deliberate revision and positive culture of drained serous fluid) or organ/space infection. LOS, rates, and causes of readmissions were also documented.

Analytical (hemoglobin, leukocytes, lymphocytes, procalcitonin, and C-reactive protein) and nutritional (total protein, albumin, prealbumin, transferrin, and zinc) variables were determined before nutritional supplementation, on the day of surgery and on the third day postoperatively.

### Ethics

The study was presented to each Hospital Ethical Board and accepted as an interventional multicenter randomized study. The research was conducted in accordance with the Helsinki Declaration and local legislation. The patients gave informed consent to participate in the study. This study has been registered in the NCT register as NCT02393976.

### Sample Size Calculation

The hypothesis of this trial was that immunonutrition would reduce the overall postoperative 30-day morbidity rate. The sample size calculation was based on the detection of significant differences in the primary endpoint parameter of the trial. We assumed a postoperative infectious complication rate of 30% in the HHS group according to several complication rates after colorectal surgery.^14–21^ With an expected complication rate of 15% in the IEF group, the trial sample size necessary for power of 80% and a 1-sided significance level of 0.05 was calculated to be 119 patients per group. An assumed 10% dropout rate in this trial (due to noncompliance, intolerance, etc.) increased the sample size to 132 patients per group. Therefore, at least 264 patients had to be included in the trial.

### Statistical Analyses

Statistical analyses of any differences between the 2 groups were performed using SPSS version 22 (SPSS Inc, Chicago, IL). The data were presented as the means ± standard deviations or as medians and interquartile ranges where appropriate. For dichotomous outcomes, the treatment groups were compared using the χ2 test. Mann–Whitney *U* and Kruskal–Wallis tests were used for continuous, non-normally distributed outcomes. For continuous, normally distributed data, ANOVA was used.

## RESULTS

### Patients

**FIGURE 1:**
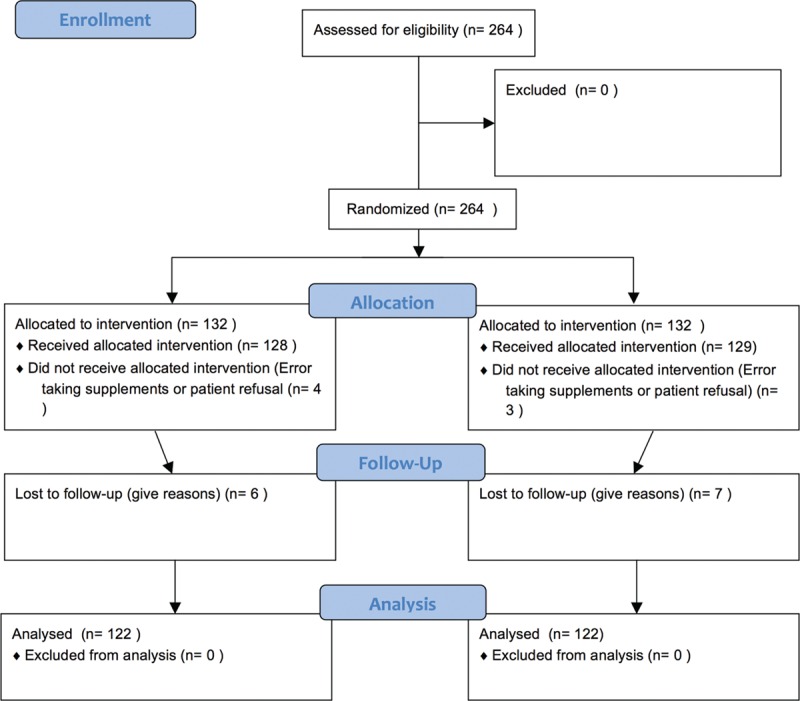
Flow diagram for the trial.

Figure 1 shows the CONSORT flowchart for the study. A total of 264 patients were randomized, 7 of whom did not receive the intervention and 13 opted not to participate after the study started. Thus, 244 of the patients who were recruited to the trial over an 18-month period (HHS, n = 122; IEF, n = 122) completed the 7-day preoperative phase of the study.

The median age of the patients was 69 years (41–89); 46.3% were women (113 patients). The patients’ ASA physical status classifications were distributed as follows: I, 14.8% (36); II, 63.9% (156); and III, 21.3% (52).

At baseline, the 2 groups were comparable for age, sex, surgical risk, comorbidity (Table 2), and analytical and nutritional parameters (Table 3).

**TABLE 2:**
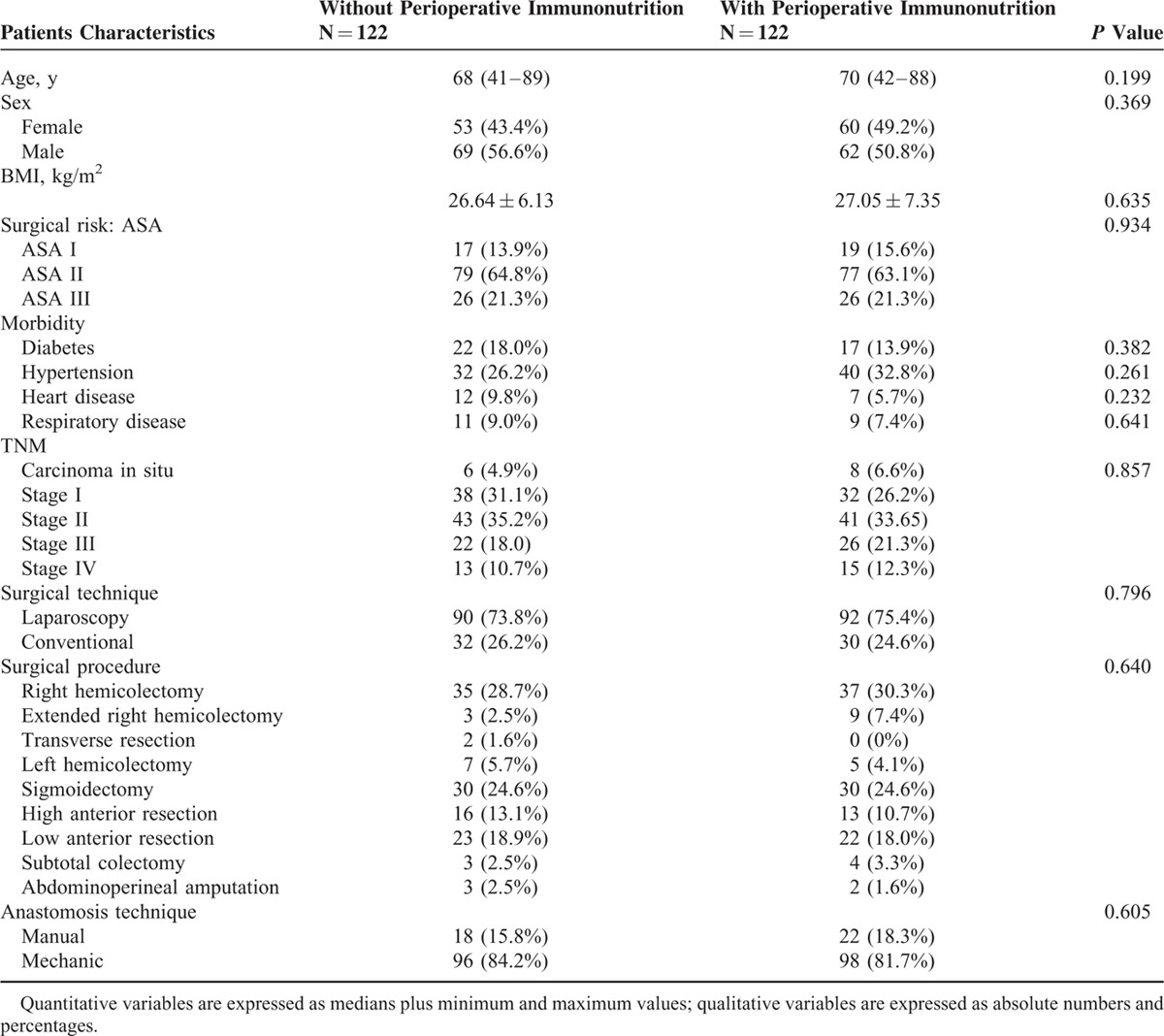
Characteristics and Surgical Procedures of the 2 Groups

**TABLE 3:**
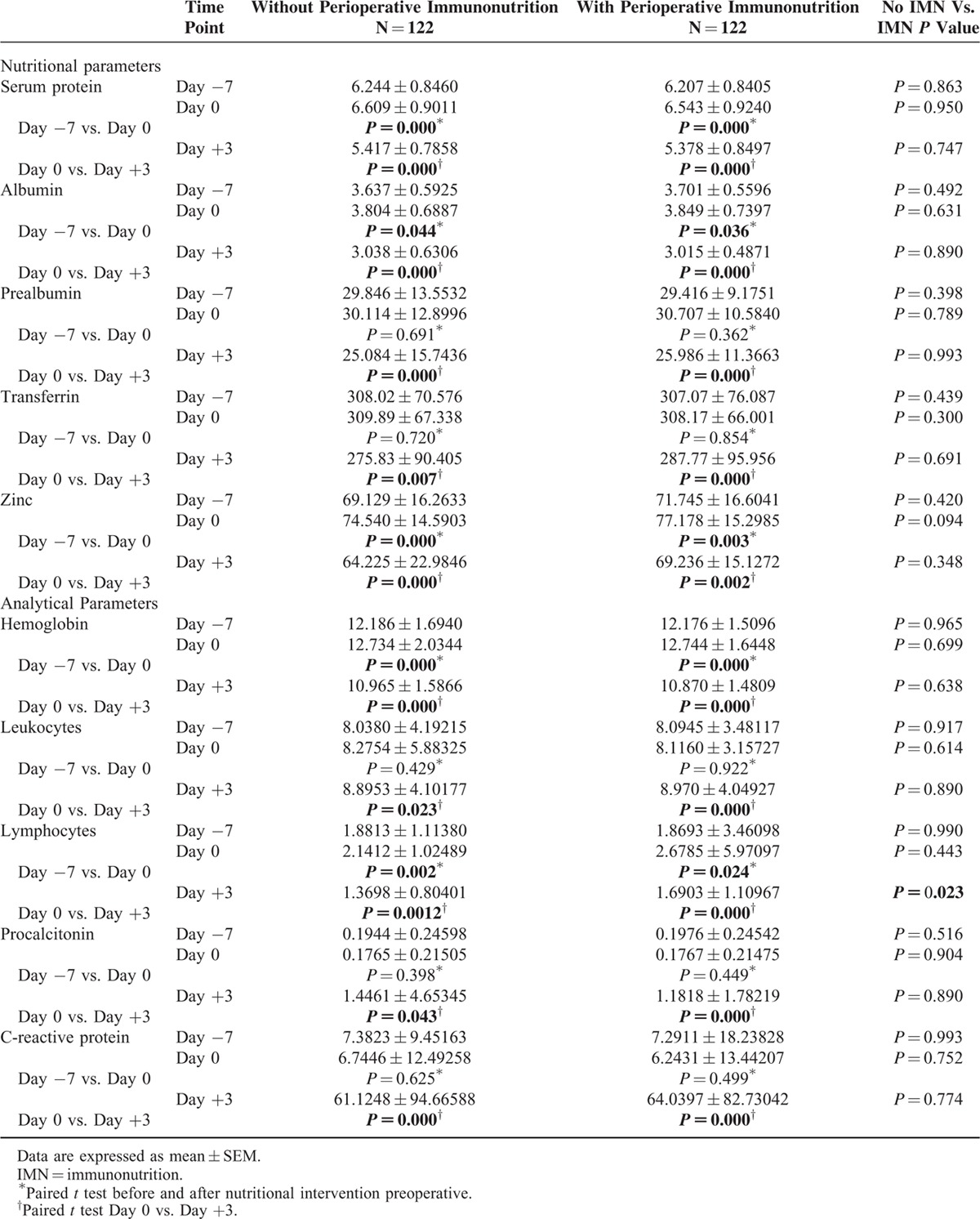
Variations of Nutritional and Analytical Parameters

### Results After 7 Days of Preoperative Nutritional Supplementation

Patient Enteral Nutritional Supplementation Compliance and Tolerance

All patients completed the preoperative nutritional treatment with a consumption of 400 mL per day. There were no differences between groups in the volume of preoperative drinks consumed. Preoperatively, the nutritional supplement drinks did not reduce the patients’ percentage of normal diet intake.

### Nutritional Laboratory Parameters

The results at baseline and after 7 days of preoperative feeding are shown in Table 3. At the time of recruitment, there were no significant differences between the 2 groups in serum protein, albumin, prealbumin, transferring, and zinc; this was also true after the nutritional treatment. However, levels of serum proteins, albumin, and zinc after the nutritional intervention were significantly higher in both groups compared with the initial determination. No significant changes in the levels of transferrin were observed after 7 days of preoperative feeding in either group (Table 3).

### Analytical Parameters

The results at baseline and after 7 days of preoperative feeding are shown in Table 3. At recruitment, there were no significant differences between the 2 groups in hemoglobin levels, leukocytes, lymphocytes, procalcitonin, or C-reactive protein; this was also true after the nutritional treatment. However, the levels of hemoglobin (ERAS includes intravenous iron supplementation in cases of preoperative anemia) and lymphocytes after the nutritional intervention were significantly higher in both groups. No significant changes in the levels of leukocytes, procalcitonin, or C-reactive protein were observed after 7 days of preoperative feeding in either group (Table 3).

### Surgery, Postoperative Treatment, and Postoperative Nutritional Supplementation

Of the patients, 74.6% (182) underwent laparoscopic surgery; 25.4% (62) underwent open surgery. Some 8.8% of the laparoscopically intervened patients required conversion to laparotomy (16). Sigmoidectomy and right hemicolectomy made up the majority of procedures performed (54.1%). The mean duration of surgery was 161.72 minutes (60–380). The median length of time spent in the recovery room was 208 minutes (60–540). There were no significant differences between the 2 groups in terms of the operative time or estimated intraoperative blood loss (121,210 mL ± 12,706 vs. 109,467 mL ± 11,804, *P* = 0,321). Table 2 shows the surgical techniques used and the surgical procedures followed.

### Main Features of the Protocol and Their Compliance Rates

Table 4 shows the compliance rates for the main features outlined in the ERAS protocol by groups. We did not find significant differences between the groups. The overall compliance to the protocol was approximately 80% but varied widely in its various components without differences between the groups.

**TABLE 4:**
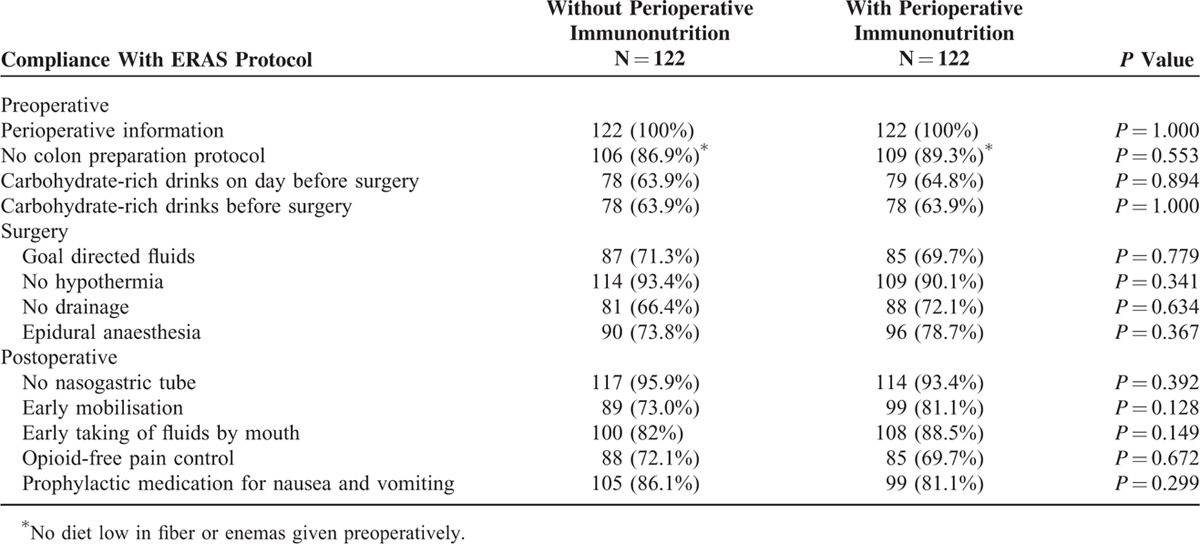
Compliance With ERAS Protocol of the 2 Groups

### Patient Enteral Nutritional Supplementation Compliance and Tolerance

After surgery, supplement intake averaged 205 ± 12.61 mL on the operative day. There were no differences between groups in the volume of postoperative drinks consumed (211 ± 12.68 mL for HHS and 198 ± 12.56 mL for IEF; *P* = 0.389).

Postoperative nutritional supplement tolerance was determined to be good in 154 patients (79 patients for IEF and 75 patients for HHS) and poor in 67 patients (34 for IEF and 33 for HHS). No supplement was ingested by 23 patients (9 and 14 patients for IEF and HHS, respectively). There were no significant differences between groups (*P* = 0.940).

Among the patients with poor tolerance, 9 in each group had nausea (27.3% in HHS and 26.5% in IEF), 31 (36.4 and 55.9%, respectively) had heaviness, 11 had heartburn (24.2 and 8.8%, respectively), and 7 had vomiting (12.1% and 8.8%, respectively). There were no statistically significant differences (*P* = 0.263).

Some 62.3% (76) for HHS and 65.6% (80) for IEF complied with the postoperative nutritional protocol (*P* = 0.594). Postoperatively, the nutritional supplements did not reduce the percentage of normal diet consumed by the patients.

### Postoperative Hospital Stay and Readmission Rate

The median length of the postoperative hospital stay was 5 days (3–52 days, 5 days for HHS (3–52) and 5 days for IEF (3–20)) with no difference between groups (*P* = 1.000). Of the patients, 3.27% (8 patients, 4 in each group) were readmitted following discharge for medical or surgical reasons without statistically significant between-group differences. Two patients presented with febrile syndrome, 3 with diarrhea, 1 with an abdominal wall abscess, 1 with a late anastomotic leak, and 1 with vomiting.

### Postoperative Morbidity/Mortality

Table 5 summarizes the complications encountered. Approximately 71% of the patients had an uneventful postoperative course without complications. As shown in the table, globally, the patients who received immunonutrition presented with fewer complications (23% vs. 35.20%, *P* = 0.035).

**TABLE 5:**
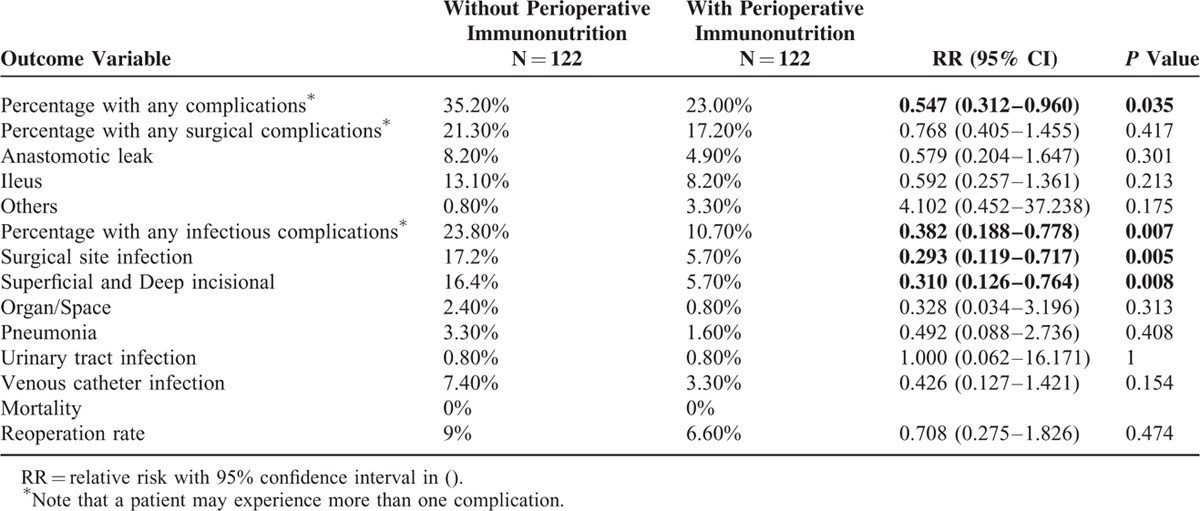
Complications

The most common complications were surgical (19.25%; 47 patients) followed by infectious complications (17.25%; 42). The most common surgical complications were paralytic ileus (10.65%; 26) and anastomosis leakage (6.55%; 16) (Table 6). Finally, the most common infectious complications were surgical site infection (superficial and deep incisional site infection (11%; 27) and organ/space infection (1.6%; 3)), urinary tract infection (0.8%; 2), and respiratory infection (2%; 5). Nineteen patients (8.8%) required repeat surgery. Causes included anastomotic leakage (16), hemoperitoneum (2), and internal hernia (1). No patients died during the hospital stay or following discharge.

**TABLE 6:**
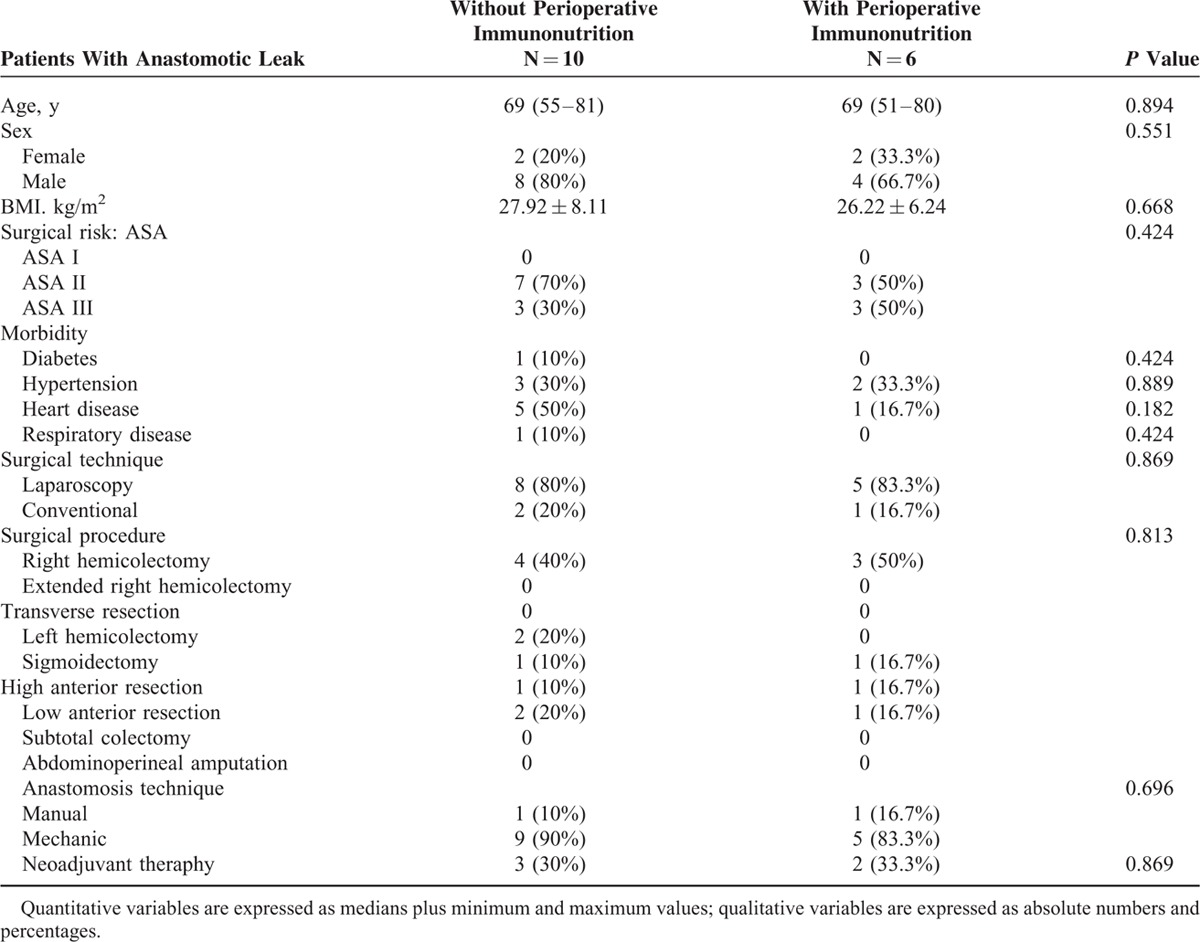
Characteristics and Surgical Procedures in Patients With Anastomotic Leaks

Table 5 shows the differences between the groups. There were fewer complications in IEF than HHS, primarily due to a significant decrease in infectious complications (23.8% vs. 10.7%, *P* = 0.0007). Among the infectious complications, surgical site infection was significantly different between groups (17.2% vs. 5.7%, *P* = 0.0005). After excluding patients with anastomotic dehiscence, surgical site infection remains higher in HHS (12.7% vs. 4.4%, *P* = 0.039). Other infectious complications were lower for IEF but without statistically significant differences. When the analysis according to the approach path is performed, we observe that, apart from this, a decrease in infectious complications occurs (*P* = 0.044 for laparoscopic surgery and *P* = 0.049 for conventional surgery).

Complications according to the Clavien-Dindo^13^ classification are shown in Table 7. To further analyze complications, we divided them into minor (Clavien-Dindo I–II) and major (Clavien-Dindo III–IV) and observed a minor complication incidence of 19.3% (for IEF and HHS, 25.4% vs. 13.1%, respectively, with statistically significant differences (*P* = 0.048)) and major complication incidence of 9.8% (9.8% vs. 9.8%).

**TABLE 7:**
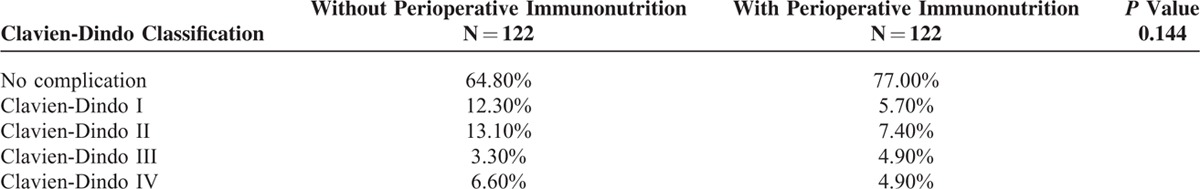
Complications According to Clavien–Dindo Classification

### Nutritional Laboratory Parameters

For both groups, the postoperative serum protein, albumin, prealbumin, transferring, and zinc levels were substantially decreased compared with the preoperative levels. The postoperative serum levels show a similar drop between groups (Table 3).

### Analytical Parameters

In both groups, postoperative serum hemoglobin levels were substantially decreased compared with preoperative levels (*P* = 0.000). Likewise, we observed an increase in white blood cell count, C-reactive protein, and procalcitonin and decreased lymphocyte count compared with their preoperative values. All of these postoperative changes were similar between the 2 groups, with the exception of lymphocyte levels in the plasma. This decrease was greater in the HHS patients, that is, IEF patients had higher levels of lymphocytes on the third postoperative day (*P* = 0.023). Postoperative levels are shown in Table 3.

## DISCUSSION

This trial showed that the combination of ERAS care and immunonutrient supplements reduces postoperative complications. Patients receiving immunonutrients preoperatively and postoperatively had fewer complications (primarily infectious) than those who received standard supplements. Notably, this study is the first to demonstrate the advantages of using immunonutrients in an ERAS protocol. Guidelines^22^ currently recommend the use of immunonutrients within these protocols; however, these recommendations have been based on scant scientific evidence. This study provides stronger evidence for this recommendation.

The first issue to be addressed when analyzing the results of our study is the use of nutritional supplements in well-nourished patients. The role of these supplements in malnourished patients is obvious; however, the role of nutritional supplements in well-nourished patients who are undergoing colorectal surgery is currently debated. Traditionally, supplementation would not be recommended for these patients; however, its use in maintaining or even improving the nutritional status of patients before surgery has spread. As our results show, supplements led to significant improvements in nutritional values (in both the IEF and HHS groups) despite being consumed for only 7 days preoperatively. Levels of serum proteins, albumin, and zinc after nutritional intervention increased even though we only include normo-nourished patients. It is therefore possible to improve the nutritional status of these patients with short-term preoperative supplementation, regardless of whether the products contain immunonutrients. Our results are consistent with a recent meta-analysis^23^ published by Hegazi et al, which recommended the use of preoperative nutritional supplements to prepare surgical patients regardless of their nutritional status.

However, surgery is a stressor on the patient and induces changes in the activity of both innate and adaptive immunity.^24^ Immune system responses after surgery can be inappropriate in some cases (e.g., systemic inflammatory response syndrome). To modulate this response, patients have recently received nutritional formulas containing certain immunonutrients, primarily arginine, glutamine, omega-3 fatty acids, and nucleotides. However, scientific evidence regarding the effectiveness of this supplementation is limited. Our results improve our knowledge of the benefits of using these substances in surgical patients.

In that same meta-analysis, no significant differences were observed between preoperative immunonutrition and standard nutrition in their effects on postoperative clinical outcomes for any type of surgery,^23^ while our results indicate otherwise. Inmunonutrition supplementation reduces global complications and infectious complications and may even, as some studies indicate, reduce anastomotic leaks.^25–27^ Perioperative administration was associated with a statistically significant reduction in anastomotic dehiscence, whereas a reduction in noninfective complications was demonstrated with postoperative administration in another meta-analysis.^28^ Our results are consistent with these meta-analyses; however, despite observing a lower incidence of anastomotic leaks, these results were not statistically significant. Notably, infectious complications were clearly reduced when the diet was supplemented with immunonutrients. However, due to the improvements in nutritional analytical values in both groups and because the best results in terms of morbidity and mortality were obtained in the immunonutrition group, we recommend that immunonutrient supplementation be provided both preoperatively and postoperatively.

Our study is one of few that focus only on patients undergoing treatment for colorectal cancer. Most existing studies include a variety of gastrointestinal surgeries. Additionally, the published results are contradictory in some cases.

For example, Braga et al^29^ demonstrated that perioperative administration of enteral supplements enriched with arginine, RNA, and omega-3 fatty acids decreases the rate of postoperative infections. The same group, which is based at the University of San Rafael in Milan, performed another interesting study comparing 4 groups: 1 group received arginine supplements and omega-3 fatty acids for 5 days preoperatively; another group, pre- and postoperative immunonutrient supplements; a third group, standard isoenergetic and isonitrogenous supplements; and a fourth group, no supplements. In this study, immunonutrition supplementation improved immune response and increased intestinal microperfusion and oxygenation. There are additional benefits to the postoperative extension of immunonutrition.^30^ Horie et al^31^ stated that preoperative immunonutrition can reduce the rate of surgical site infection.

However, not all published results find benefits of immunonutrients in terms of postoperative infection. Helminen et al^32^ observed no benefit for routinely prescribed immunonutrition. Sorensen et al^33^ (elective surgery for colorectal cancer) and Finco et al^34^ (laparoscopic colorectal surgery) reached the same conclusion.

Another important aspect of our study is the less marked reduction in lymphocyte values experienced by the immunonutrition group postoperatively. As the only value with statistically significant differences in both groups, we can assume that the existing difference in postoperative complications between the groups may result from these values. In fact, it has recently been reported that a shift toward Th2 dominance in the Th1/Th2 cytokines during the early postoperative period is directly associated with infectious complications.^35^ Matsuda et al^36^ described a correction in the balance of Th1/Th2 cytokines in patients with colorectal cancer who were undergoing surgery and received an oral diet supplemented with arginine, omega-3 fatty acids, and ribonucleic acid. They concluded that this balance correction may be an important determinant of the clinical benefits of immunonutrients and of how immunonutrition reduces postsurgical infections.

The degree of compliance with the items included in the ERAS program is an important factor in the reduction of postoperative complications and the achievement of early recovery of our patients. In our study, compliance was higher than 80%. If we compare these results with those recently published by The ERAS Compliance Group^37^ (overall compliance of 76.6% for colon cancer and 75.0% for rectal cancer), the compliance rates are very similar and much higher than those published when we started these programs.^10^ It is therefore shown that compliance increases as the teams gain experience in these protocols.

Although ERAS reduces the risk of complications compared with the traditional treatment scheme^38^ and each of the steps outlined in ERAS is based on scientific evidence, it is necessary to continue working on methods to reduce complications. Immunonutrition represents one of these potential points of entry.

Our study has some limitations. First, the number of cases was insufficient to make robust conclusions and the sample size has been calculated to obtain a statistically significant difference for infectious complications. We suggest a study with more participants to check if noninfectious complications also decreased when inmunonutientes enriched supplements are used. Second, not all patients could ingest all postoperative supplements, although there were no differences in intake between the 2 groups. Likewise, although similar in composition (except immunonutrients), there is a global difference of approximately 100 kcal per day between the 2 nutritional supplements, we do not believe that influence, as well as this dietary supplement was consumed in addition to normal food intake. Third, not being double-blind study could contribute to some form of bias. On the other hand, our study was performed at 6 reference centers in Spain with multidisciplinary teams who were fully dedicated to colorectal surgery and had previous experience in the implementation of ERAS programs. Therefore, it may be difficult to reproduce our results in nonexperienced groups. Finally, despite using 2 comparable groups, we included various surgical procedures and 2 modalities (laparoscopic and conventional).

## CONCLUSIONS

Based on the data from the present multicenter, randomized study, the implementation of ERAS protocols including immunonutrient-enriched supplements reduces complications in patients undergoing colorectal resection. However, further studies are needed to understand how immunonutrients improve the prognosis of patients with colorectal cancer and the potential mechanisms involved in immunonutrient-enriched supplements reduces the complications of patients undergoing colorectal resection.
